# Dimming Titan Revealed by the Cassini Observations

**DOI:** 10.1038/srep08239

**Published:** 2015-02-04

**Authors:** Liming Li

**Affiliations:** 1 Department of Physics, University of Houston, Houston, TX 77204, USA

## Abstract

Here we report the temporal variation of Titan's emitted energy with the Cassini/CIRS observations. In the northern hemisphere, the hemispheric-average emitted power decreased from 2007 to 2009 and increased from 2009 to 2012–13, which make the net change insignificant (0.1 ± 0.2%) during the period 2007–2013. The decrease from 2007 to 2009 is mainly due to the cooling around the stratospause, and the increase from 2009 to 2012–13 is probably related to temporal variation of atmospheric temperature around the tropopuase in the northern hemisphere. In the southern hemisphere, the emitted power continuously decreased by 5.0 ± 0.6% from 2.40 ± 0.01 W/m^2^ in 2007 to 2.28 ± 0.01 in 2012–13, which is mainly related to Titan's seasonal variation. The asymmetry in the temporal variation between the two hemispheres results in the global-average emitted power decreasing by 2.5 ± 0.6% from 2.41 ± 0.01 W/m^2^ in 2007 to 2.35 ± 0.01 W/m^2^ in 2012–13. The solar constant at Titan decreased by ~13.0% in the same period 2007–2013, which is much stronger than the temporal variation of emitted power. The measurements of Titan's absorbed solar power are needed to determine the temporal variation of the global energy budget.

The emitted thermal energy and the absorbed solar energy determine the energy budget at the top of atmosphere of a planet or satellite, which further sets boundary conditions for the atmospheric system. The transfer and distribution of the radiant energies inside of the atmospheric system modify the thermal structure to generate available potential energy. The available potential energy can be further converted into kinetic energy to drive atmospheric circulation and the related weather and climate on the astronomical body. For a basically equilibrium climate like the current one on our home-planet Earth, the two energy components (i.e., the emitted thermal energy and the absorbed solar energy) are roughly balanced^1^,^2^, even though there are small fluctuations^3^,^4^,^5^,^6^ with a magnitude of ~0.1% in the global-average energy components.

The temporal variations of Earth's energy budget and their implications for climate change have already been extensively investigated in recent years^4^,^5^,^6^,^7^. However, we know little about the temporal variations of the global energy budgets of other terrestrial bodies. Titan, the biggest satellite of Saturn, is a terrestrial body similar to Earth in many aspects^8^. The Cassini spacecraft has already conducted in-orbit observations of Saturn's system including Titan since 2004, and hosts 12 scientific instruments sensitive to various wavelengths. Among them, the Composite Infrared Spectrometer (CIRS)^9^ records infrared spectra with a wide coverage of wavelengths, which include spectral bands mainly contributing to the emitted thermal energy. The Cassini/CIRS observations have already been used to examine the global emitted thermal energies of some astronomical bodies^10^,^11^,^12^. In this paper, we explore the temporal variation of Titan's emitted power, which is one component of its global energy budget, with the long-term Cassini/CIRS observations. The methodology of computing the emitted power has already been introduced in some classical studies^13^,^14^,^15^,^16^ and used in our previous studies^10^,^11^,^12^. The basic idea is to integrate the thermal spectra over both latitude and emission angle to get the total emitted thermal energy from a planet or a satellite. The thickness of Titan's atmosphere is also considered by projecting the original CIRS observations from the surface to a reference altitude of 500 kilometers to capture all emitted energy from Titan^11^.

## Results

The CIRS observations generally have good coverage in both latitude and emission angle, but there are significant gaps (especially in the polar regions) in some years that make it impossible to explore Titan's global emitted power. By examining the CIRS observational coverage, we select three years (2007, 2009, and 2012) with good coverage in latitude and emission angle to compute Titan's global emitted power. In the case of data from 2012, we also include the public data in the first half of 2013 to improve the spatial coverage.

**Figure 1:**
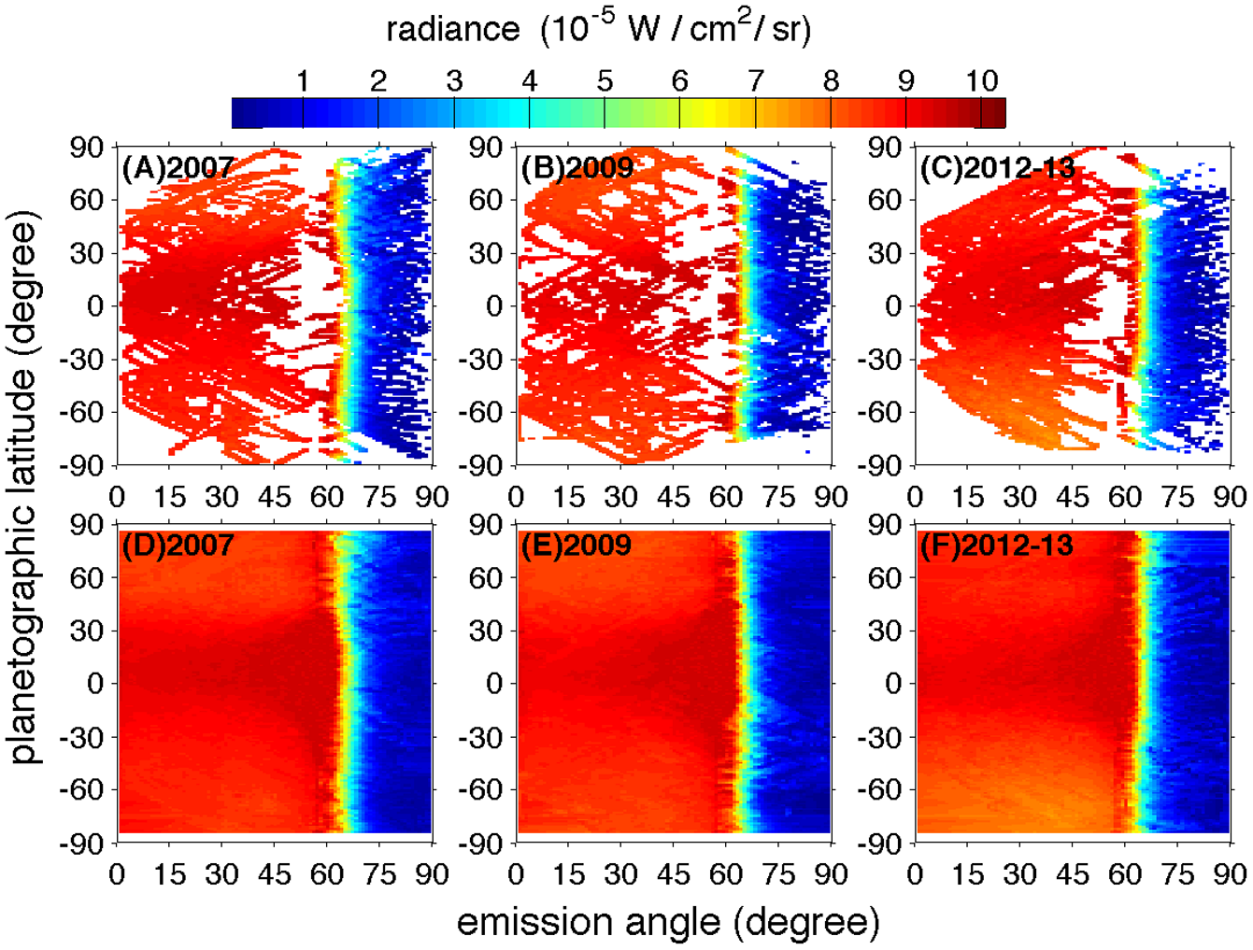
Coverage of the CIRS observations at the reference altitude of 500 kilometers. The wavenumber-integrated radiance in the plane of latitude and emission angle is averaged in 2007 (panel A), 2009 (panel B). Panel C is a time average over 2012 and the first half year of 2013. The radiance with emission angle less than 57° at the reference altitude (500 kilometers) comes from CIRS nadir observations, and the radiance with emission angle larger than 57° comes from CIRS limb observations. Panels (D), (E), and (F) are same as panel (A), (B), and (C) except for filling the observational gaps with the least squares fitting.

**Figure 2:**
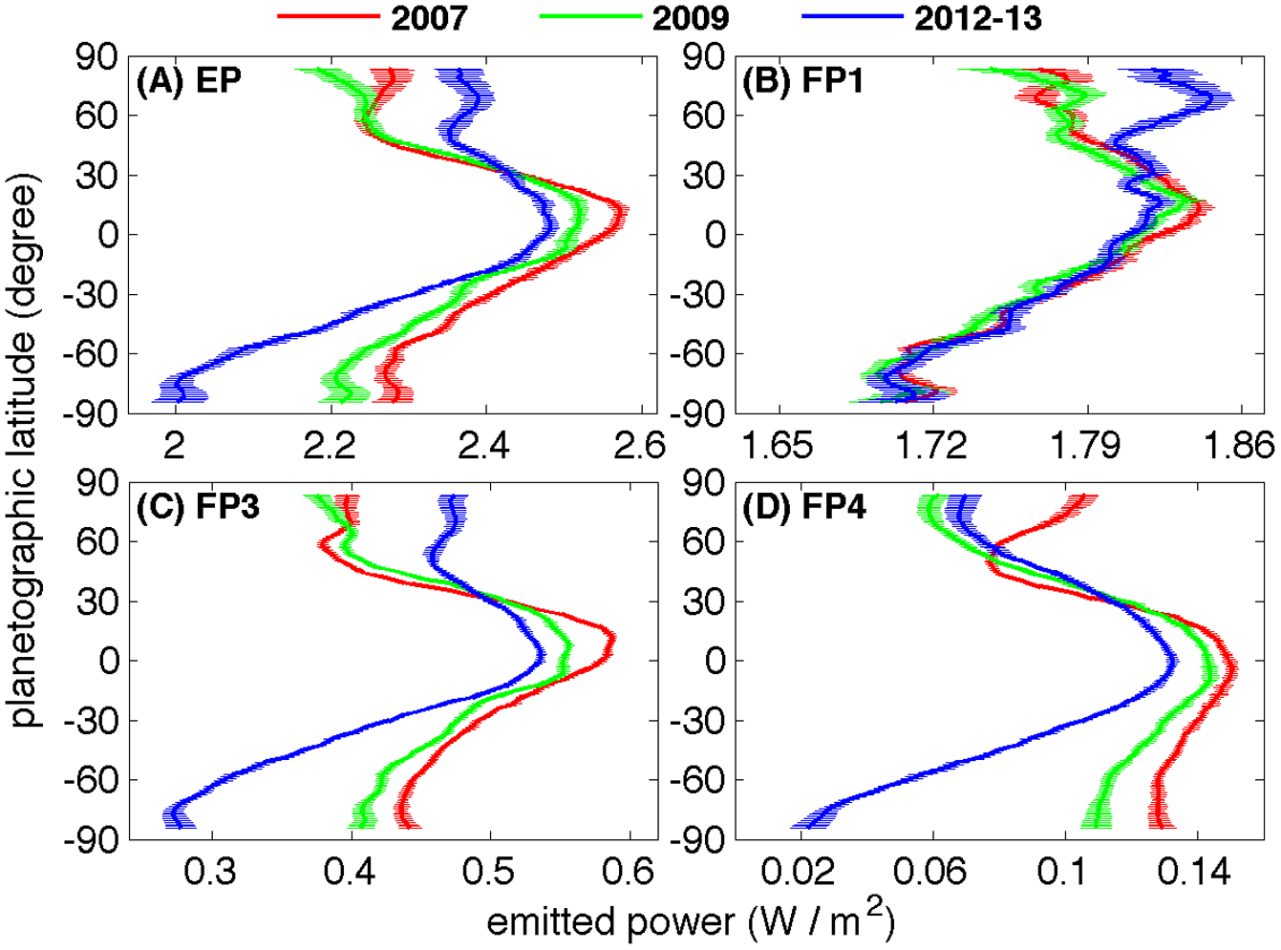
Meridional distribution of Titan's emitted power. (A) Total emitted power. Panels (B), (C), and (D) are thermal radiance recorded by the focal planes FP1, FP3, and FP4, respectively. The thick line is the profile of the emitted power and horizontal lines represent the uncertainties. The estimated uncertainty is combined by the uncertainty related to the filling observational gaps and the uncertainty related to the CIRS data calibration.

The sub-solar latitudes for the selected years are shown in Table 1. Table 1 also includes the solar longitude, which is defined as the angular distance along Titan's orbit around the Sun measured from northern spring equinox (0°). The coverage of CIRS observations in the directions of latitude and emission angle in the selected years is shown in Fig. 1 (Panels A, B, and C). Figure 1 shows that there are still some observational gaps, which are filled by linear interpolation used in our previous studies^10^,^11^,^12^. The coverage after filling the observational gaps is shown in panels D, E, and F of Fig. 1. After filling the observational gaps, we can integrate the thermal radiance in the direction of emission angle to get the emitted power at each latitude, which is shown in panel A of Fig. 2. The uncertainty in Fig. 2 is estimated by combining the error sources from filling observational gaps and the CIRS data calibration, as we discussed in the previous studies^10^. Panel A shows that there are significant temporal variations of emitted power from 2007 to 2013 at most latitudes, which are larger than the corresponding uncertainty. Titan's emitted power continuously decreased from 2007 to 2013 not only in the southern hemisphere (SH) but also in the low latitudes (0–30°N) of the northern hemisphere (NH). In particular, the emitted power decreased by 12.1 ± 1.7% from 2.28 ± 0.02 W/m^2^ in 2007 to 2.00 ± 0.03 W/m^2^ in 2012–13 in the southern polar region (75–85°S). In the mid southern latitudes (30–60°S) and the tropical region (30°N–30°S), Titan's emitted power decreased 6.3 ± 0.8% and 2.9 ± 0.6% respectively during the period of 2007–2013. In the high latitudes (45–85°N) of the NH, Titan's emitted power increased ~4.8 ± 1.1% from 2007 to 2012–13.

In order to understand the temporal variation of emitted power, we further compute the emitted power at different wavelengths. The Cassini/CIRS has three focal planes: FP1 (10–695 cm^−1^), FP3 (570–1125 cm^−1^), and FP4 (1025–1430 cm^−1^). To decrease the CIRS observational noise around the ends of the wavenumber interval covered by each focal plane, we choose wavenumber ranges 10–600 cm^−1^ for FP1, 600–1050 cm^−1^ for FP3, and 1050–1430 cm^−1^ for FP4 to compute Titan's emitted power. It is possible to compute the emitted power by organizing the spectra in narrower spectral ranges than the wavenumber range covered by each focal plane, but the thermal radiance averaged over a narrower wavenumber range generally has more noise and larger uncertainty. Here, we integrate the thermal radiance over wavelengths covered by each of the three focal planes to compute the emitted power. The inversion kernels of temperature soundings of Titan by the Cassini/CIRS^9^ suggest that the radiances at a few FP1 wavenumbers (e.g., 15 cm^−1^, 60 cm^−1^, and 90 cm^−1^) emit from the pressure levels around Titan's tropopause (i.e., ~100 mbar). The FP1 also includes a wavenubmer (i.e., 530 cm^−1^), in which the surface radiance can escape to space^17^,^18^,^19^. The radiances at most FP3 wavenumbers emit from the middle stratosphere, and the radiances at most FP4 wavenumbers emit from the upper stratosphere and lower mesosphere^9^.

**Figure 3:**
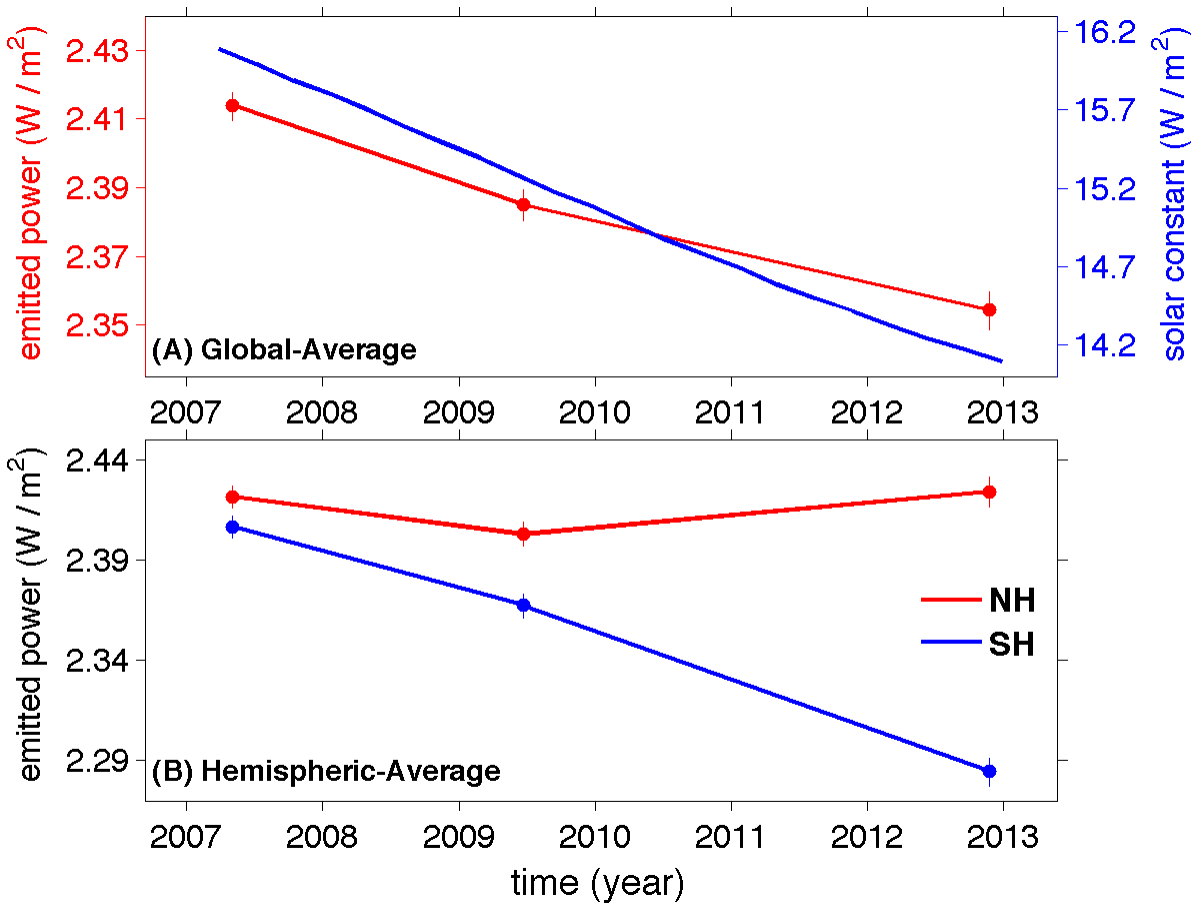
Temporal variation of global/hemispheric-average emitted power during the period of 2007–2013. (A) Global-average emitted power and the solar constant at Titan. (B) Hemispheric-average emitted power. The times of points are the average observational times over the selected years. The error bars are estimated by combining the uncertainty related to the CIRS data calibration and filling observational gaps.

The thermal radiance recorded by each of the three CIRS focal planes is shown in panels B, C, and D of Fig. 2. The continuing decrease of Titan's total emitted power from 2007 to 2013 in the middle and high latitudes of the SH (30–85°S) and the tropical region (30°N–30°S), which is visible in panel A of Fig. 2, originated mainly in the wavenumber intervals of focal planes FP3 and FP4 (panels C and D). Therefore, the cooling in the stratosphere and lower mesosphere, which are recorded by FP3/4, contributes to the temporal variation of emitted power from 2007 to 2013 in the SH and the tropical region. The continuing decrease of emitted power in the tropical region from 2007 to 2013 is probably related to the decreased solar constant, which is shown in panel A of Fig. 3. In the middle and high latitudes of the SH, the continuous decrease of emitted power is also related to the seasonal change of Saturn, in which the sub-solar latitude changed from the SH to the NH during the period 2007–2013 (Table 1). The radiative time constant is relatively short in Titan's middle stratosphere observed by FP3 (~a few Earth years) and the upper stratosphere by FP4 (less than 1 Earth year)^20^,^21^. The seasonal change of solar radiance and the short radiative time constant of the upper atmosphere are consistent with the decreased emitted power in the tropical region and the SH.

In the middle and high latitudes of the NH (45–85°N), the increase of emitted power from 2009 to 2013 (panel A) is mainly contributed by the temporal variation shown in the FP1 (panel B) and FP3 (panel C). In particular, the increase of thermal radiance from 2009 to 2013 shown by FP1 (panel B) implies that the atmospheric temperature around the tropopause probably experienced warming in the middle and high latitudes of the NH. The increase of emitted power in the middle and high latitudes of the NH happened mainly from 2009 to 2013, which lags the increase of solar flux in the NH since 2002 when the sub-solar latitude began to move northward. Such a lag is possibly due to the relatively long radiative time constant around the tropopause (~50 Earth years) and middle stratosphere (~a few Earth years)^20^,^21^. It is also possible that other factors (e.g., heat transport by the atmospheric circulation^22^) contribute to the temporal variation of emitted power in the middle and high northern latitudes. In the northern polar region (60–85°N), the decrease of emitted power from 2007 to 2009 (panel A of Fig. 2) is mainly due to a cooling around the stratospause around 0.1 mbar, which was measured by a previous study^22^ and panel D of Fig. 2 (FP4). In summary, the temporal variation of emitted power is more complicated in the NH than in the SH. The solar constant at Titan decreased from 2007 to 2013 due to the increased Sun-Titan distance (Fig. 3). At the same time, the seasonal change in which the sub-solar latitude moved from the SH to the NH helped to increase the solar irradiance in the NH during the period 2007–2013 (Table 1). The combined effects make the temporal variation of atmospheric temperature complicated, which contributes to the different behaviors of emitted power in the different latitudes of the NH. In addition, atmospheric circulation also helps modify the atmospheric temperature and hence the emitted power^22^,^23^.

Assuming the emitted power in the latitude band of 86–90°N/S, in which the observations are too few to get measurements of emitted power, has the same value and uncertainty as the value at 85°S/N, we can integrate the profile of emitted power in the meridional direction to get the global-average emitted power. The observational gaps in the polar regions do not significantly contribute to the uncertainty of the global-average emitted power, because the area covered by the latitudinal band of 86–90°N/S occupies only ~0.3% of the global area. Panel A of Fig. 3 suggests that the global-average emitted power decreased by 2.5 ± 0.6% from 2.41 ± 0.01 W/m^2^ in 2007 to 2.35 ± 0.01 W/m^2^ in 2012–13. Titan is in the mid northern winter and mid northern spring in 2007 and 2012–13, respectively (Table 1). Therefore, the large temporal variation from 2007 to 2012–13 suggests that Titan's global-average emitted power varies at least 2.5% at the timescale of one season. On Earth, the seasonal cycle of emitted power is very small at a magnitude of 0.1%^3^,^4^. Therefore, the temporal variation of global emitted power seems to be significantly larger on Titan than on Earth. Titan's hemispheric-average emitted power is also computed, which is shown in panel B of Fig. 3. In the NH, Titan's emitted power decreased from 2007 to 2009 and increased roughly after the northern spring equinox (August, 2009), so the net change from 2007 to 2013 is 0.003 ± 0.006 W/m^2^ (~0.1 ± 0.2% of the emitted power). Therefore, the net change from 2007 to 2013 is not significant with the uncertainty larger than the variation. On the other hand, the emitted power in the SH continuously decreased from 2.40 ± 0.01 W/m^2^ in 2007 to 2.28 ± 0.01 in 2012–13. The temporal variation of SH-average emitted power is 5.0 ± 0.6% during the time period of 2007–2013. Therefore, the hemispheric-average emitted power displays stronger temporal variation in the SH than in the NH during the period of 2007–2013.

## Discussion

This study examines the temporal variation of Titan's emitted power. The meridional profiles of emitted power suggest that the temporal variation of emitted power behaves differently at different latitudes. In particular, the seasonal change of emitted power is different between the two hemispheres during the period of 2007–2013. Our study also suggests that Titan's global-average emitted energy significantly decreased by 2.5 ± 0.6% from 2007 to 2013. The extended Cassini observations from 2014 to 2017 will further help us examine if the trend of decreasing global emitted power will continue.

Titan's radiant energy budget is determined by the emitted power and the absorbed solar power. The latter is further determined by the solar flux at the top of the atmosphere (i.e., solar constant) and the Bond albedo. Saturn has the large orbital eccentricity (~0.057), which results in a large variation of Sun-Titan distance at the seasonal scale. Therefore, the solar constant decreased by ~13% from 16.1 W/m^2^ in 2007 to 14.0 W/m^2^ in 2013 (panel A of Fig. 3). Titan's Bond albedo must be measured to determine the absorbed solar power with the known solar constant. Previous studies suggest that Titan's brightness and hence albedo displayed north-south asymmetry^24^ and temporal variation^25^,^26^,^27^. The temporal variation of Titan's brightness and albedo is mainly due to the temporally-varying hazes and clouds^25^,^26^,^27^,^28^,^29^,^30^. These previous observations of seasonal photometric variability are limited to a few phase angles and wavelengths^25^,^26^,^27^. The solar energy mainly comes from the spectral range of ~0.1–3.0 μm. Therefore, the precise measurements of Titan's Bond albedo and its temporal variation require observations covering the whole spectral coverage from 0.1 μm to ~3.0 μm, because the reflection of solar radiance displays different temporal behaviors at different wavelengths^25^,^26^. In addition, the measurements of Bond albedo require observations with the coverage of phase angle from 0° to 180°. The observations recorded by the Imaging Science Subsystem and the Visual and Infrared Mapping Spectrometer onboard the Cassini spacecraft basically provide such observations. We are processing the Cassini observations to measure Titan's Bond albedo. Then we can determine the absorbed solar energy, which will be combined with this study to investigate Titan's global energy budget and its temporal variation.

Some recent studies of Earth's energy budget suggest that Earth is experiencing a small energy imbalance with the absorbed solar energy greater than the emitted thermal energy^4^,^5^,^6^. The small energy imbalance at the top of Earth's atmosphere significantly contributes to climate change, adding to the effects from greenhouse gases^5^,^6^. It will be interesting to investigate the effects of the possible energy imbalance, if discovered, on the temporal evolution of Titan's atmosphere besides the greenhouse effects (e.g., H_2_ and CH_4_) and antigreenhouse effects (e.g., high-altitude haze)^8^.

## Author Contributions

L.L. conducted all aspects of the manuscript.

## Figures and Tables

**Table 1:** Selected Cassini/CIRS observations and the corresponding solar longitudes and sub-solar latitudes

Observational Time	2007	2009	2012–13
**Solar Longitude**	330.3°	358.1°	39.0°
**Sub-solar Latitude**	12.3°S	0.6°S	16.7°N
